# Cytological and transcriptome analyses reveal *OsPUB73* defect affects the gene expression associated with tapetum or pollen exine abnormality in rice

**DOI:** 10.1186/s12870-019-2175-2

**Published:** 2019-12-10

**Authors:** Lin Chen, Ruilian Deng, Guoqiang Liu, Jing Jin, Jinwen Wu, Xiangdong Liu

**Affiliations:** 1 State Key Laboratory for Conservation and Utilization of Subtropical Agro-Bioresources, South China Agricultural University, Guangzhou, 510642 China; 2 Guangdong Provincial Key Laboratory of Plant Molecular Breeding, South China Agricultural University, Guangzhou, 510642 China; 3 Guangdong Laboratory for Lingnan Modern Agriculture, South China Agricultural University, Guangzhou, 510642 China; 4 Vegetable Research Institute, Guangdong Academy of Agricultural Sciences, Guangzhou, 510640 China; 5 Guangdong Key Laboratory for New Technology Research of Vegetables, Guangzhou, 510640 China

**Keywords:** Rice, Ubiquitin ligase activity, Transcriptome, Male reproductive development

## Abstract

**Background:**

As one of the main crops in the world, sterility of rice (*Oryza sativa* L.) significantly affects the production and leads to yield decrease. Our previous research showed that *OsPUB73*, which encodes U-box domain-containing protein 73, may be associated with male sterility. However, little information is available on this gene that is required for anther development. In the present study, we knocked out *OsPUB73* by using the CRISPR/Cas9 system and studied the cytological and transcriptome of the gene-defect associated with pollen development and sterility in the rice variety (Taichung 65).

**Results:**

The sequence analysis indicated that *OsPUB73* was comprised of 3 exons and 2 introns, of which CDS encoded 586 amino acids including a U-box domain. The expression pattern of *OsPUB73* showed that it was highly expressed in the anther during meiosis stage. The *ospub73* displayed low pollen fertility (19.45%), which was significantly lower than wild type (WT) (85.37%). Cytological observation showed tapetum vacuolated at the meiosis stage and pollen exine was abnormal at the bi-cellular pollen stage of *ospub73*. RNA-seq analysis detected 2240 down and 571 up-regulated genes in anther of *ospub73* compared with WT during meiosis stage. Among of 2240 down-regulated genes, seven known genes were associated with tapetal cell death or pollen exine development, including *CYP703A3* (*Cytochrome P450 Hydroxylase703A3*), *CYP704B2* (*Cytochrome P450 Hydroxylase704B2*), *DPW* (*Defective Pollen Wall*), *PTC1* (*Persistant Tapetal Cell1*), *UDT1* (*Undeveloped Tapetum1*), *OsAP37* (*Aspartic protease37*) and *OsABCG15* (*ATP binding cassette G15*), which were validated by quantitative real-time polymerase chain reaction (qRT-PCR). These results suggested *OsPUB73* may play an important role in tapetal or pollen exine development and resulted in pollen partial sterility.

**Conclusion:**

Our results revealed that *OsPUB73* plays an important role in rice male reproductive development, which provides valuable information about the molecular mechanisms of the U-box in rice male reproductive development.

## Background

Rice (*Oryza sativa* L.) is one of the most essential agricultural crops and feeds over half of the global population. Improving the productivity of rice grain is necessary for food security. However, low seed setting is a major hindrance in the rice yields; moreover male reproductive development presents correlation with success in seed setting.

It is well known that male reproductive development is a critical biological process involving the generation of haploid pollen for sexual reproduction; and anther development is the principal event in male reproductive development [1, 2]. The anther is comprised of a four-lobed structure, and each lobe includes microsporocytes, the epidermis, endothecium, middle layer, and tapetum after the morphogenesis. The tapetum is the innermost cell layer of the anther and provides a safe surrounding, necessary nutrients and enzymes during microspore development [3]. The tapetum begins to degenerate after the meiosis, which is considered to be a process of programmed cell death (PCD). The tapetum degradation promotes the formation of pollen walls and releases microspores. Normal tapetum degradation is critical for the production of viable pollen grains in the male reproductive development, and abnormal tapetum degradation usually causes pollen sterility [4–6]. In the previous studies, some genes controlling tapetum development have been found in rice [7–14]. A MYB transcriptional factor (GAMYB) has been considered important for pollen development, and *gamyb* mutants displayed abnormal development of exine and Ubisch bodies [15, 16]. Li et al. [17] reported that *PTC1* encodes a PHD-finger protein that controls tapetal degeneration, pollen wall formation, and aborted microspores.

The ubiquitin–proteasome system, which is involved in post-translational modification, is a key regulatory mechanism for plant growth and development [18, 19]. The ubiquitin–proteasome system involves three essential enzymes, including ubiquitin-activating enzyme (E1), ubiquitin-conjugating enzyme (E2), and ubiquitin ligase (E3) [20]. The E3 ligase plays an important role in the regulation of the ubiquitin–proteasome system and confers specificity to the ubiquitination reaction. E3 ligases modify a large number of proteins or protein complexes, most of which contain RING, HECT, F-box, and U-box domains [21–24]. The U-box, which contains about 70 amino acids, is a highly conserved domain and the E3 ligase activity-related protein domain; and was first shown to be involved in polyubiquitin chain assembly in yeast [25, 26]. A number of predicted plant U-box (PUB) family proteins in rice and *Arabidopsis thaliana* can be classified into nine groups according to their other distinguishing domains, including UFD2 specific motif+U-box, U-box+ARM/HEAT, U-box+GKL-box, Kinase+U-box, U-box only, U-box+WD40, TPR + U-box, TPR + Kinase+U-box and MIF4G + U-box, and the U-box+ARM/HEAT is the largest group [27]. PUB proteins are involved in various cellular processes in higher plants, including abiotic stress responses [28], plant hormone regulation [29, 30], flowering time [31], cell division and elongation, plant cell death and defense responses [32, 33]. In addition, *Atpub4* showed incomplete degeneration of tapetal cells and their pollen grains had abnormal exine structure. These results indicate that PUB family proteins also play an important role in the plant male fertility.

*OsPUB73* encodes a U-box protein and possesses E3 ligase activity in rice [27]. Our previous research showed that *OsPUB73* was down-regulated in autotetraploid rice hybrid of multi-allelic interactions at pollen sterility loci compared to corresponding diploid rice hybrid by transcriptome analysis, suggesting that *OsPUB73* may be associated with male sterility in rice [34]. To investigate the molecular mechanism of *OsPUB73*, we developed *ospub73* by using the CRISPR/Cas9 technology. Cytological observation was used to investigate the fertility of *ospub73* and WT, and we primarily aimed to evaluate the role of *OsPUB73* for male reproductive development. In addition, transcriptome analysis of anther was carried out to identify DEGs (differentially expressed genes) between *ospub73* and WT during meiosis. Our study provides an important evidence of the role of PUB in regulating male reproductive development.

## Results

### Sequence analysis and expression pattern of *OsPUB73*

**Fig. 1:**
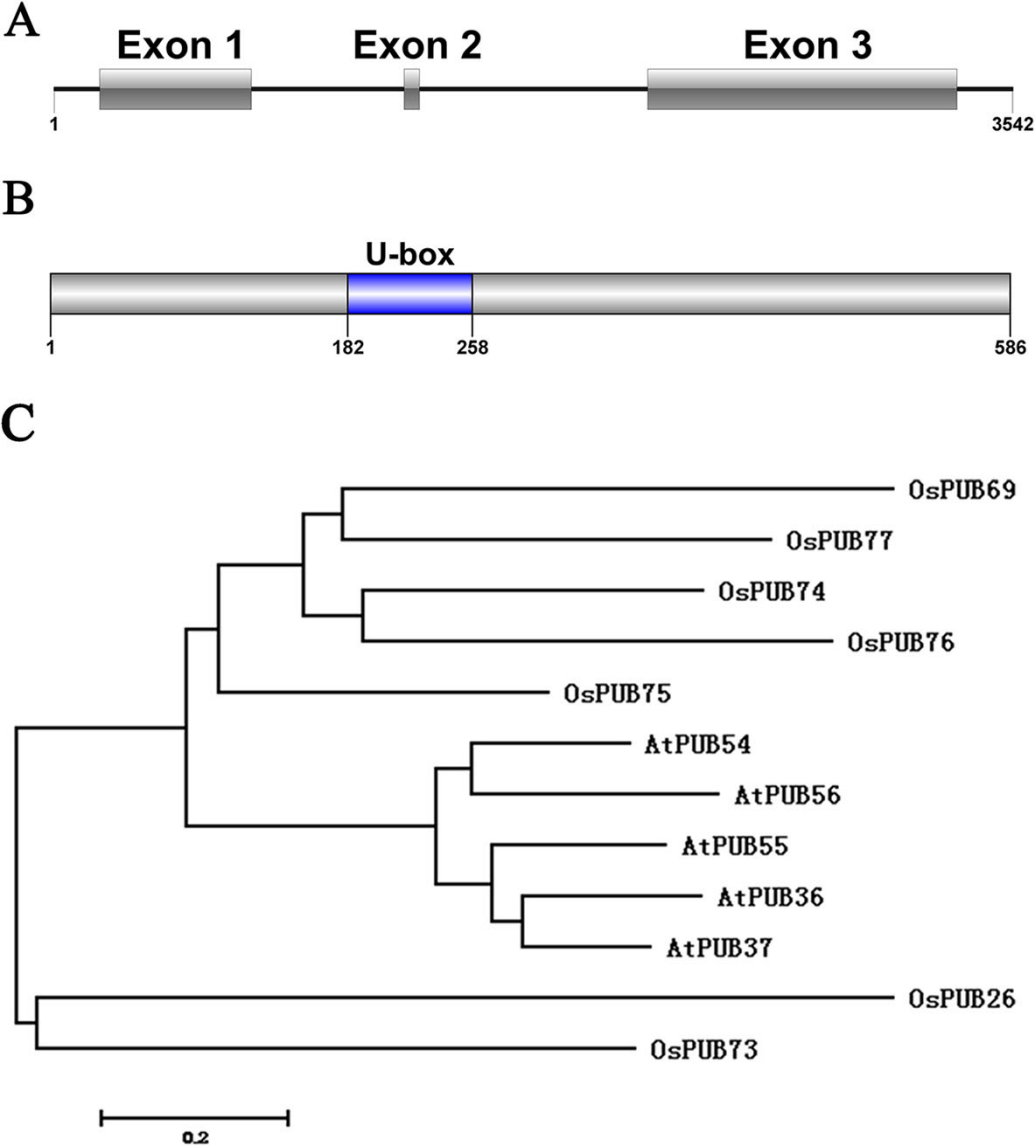
The genome structure and phylogenetic analysis. **a** The genome structure of *OsPUB73* in rice genome; **b** The site of U-box structure in *OsPUB73*; C, Comparative phylogenetic analysis of *OsPUB73* protein in rice and *arabidopsis*, the evolution history was inferred using Neighbor-Joining phylogenetic tree generated with the MEGA6.0

The sequence of *OsPUB73* didn’t show any variation in the Taichung-65 compared to Nipponbare by re-sequencing, and was consistent with the full-length sequence from the Rice Genome Annotation Project Database (http://rice.plantbiology.msu.edu/). The *OsPUB73* comprised three exons and two introns (Fig. 1a). The CDS sequence of *OsPUB73* is 1758 bp (Additional file 1: Figure S1), and it encodes ubiquitin ligase activity-related protein of 586 amino acids, which includes a U-box domain (Fig. 1b). Zeng et al. [27] found 77 and 63 genes encoding U-box domain-containing proteins in the rice and *Arabidopsis* genomes, respectively. The *OsPUB73* belonged to V Class (U-box only). To investigate the phylogenetic relationship of V Class between rice and *Arabidopsis*, 12 genes (included 7 V Class in rice and 5 V Class in *Arabidopsis*) were used for sequence comparison and phylogenetic analysis. *OsPUB73* and *OsPUB26* were found to be an orthologous pair (Fig. 1c), and U-box domain was detected in all genes (Additional file 2: Figure S2).

The RT-PCR (rverse transcription polymerase chain reaction) and qRT-PCR analysis were used to survey the spatial and temporal patterns of *OsPUB73* in Taichung-65 plants. The *OsPUB73* expression was mainly identified in anthers during meiosis stage, and the expression of *OsPUB73* was almost undetectable in the mature anthers. In addition, trace amounts of *OsPUB73* expression were also detected in the roots, stems and leaves (Additional file 3: Figure S3). The high levels of *OsPUB73* expression in meiosis stage anthers are consistent with its role in regulating male reproductive development.

### Creation of *ospub73* using the CRISPR/Cas9 system

**Fig. 2:**
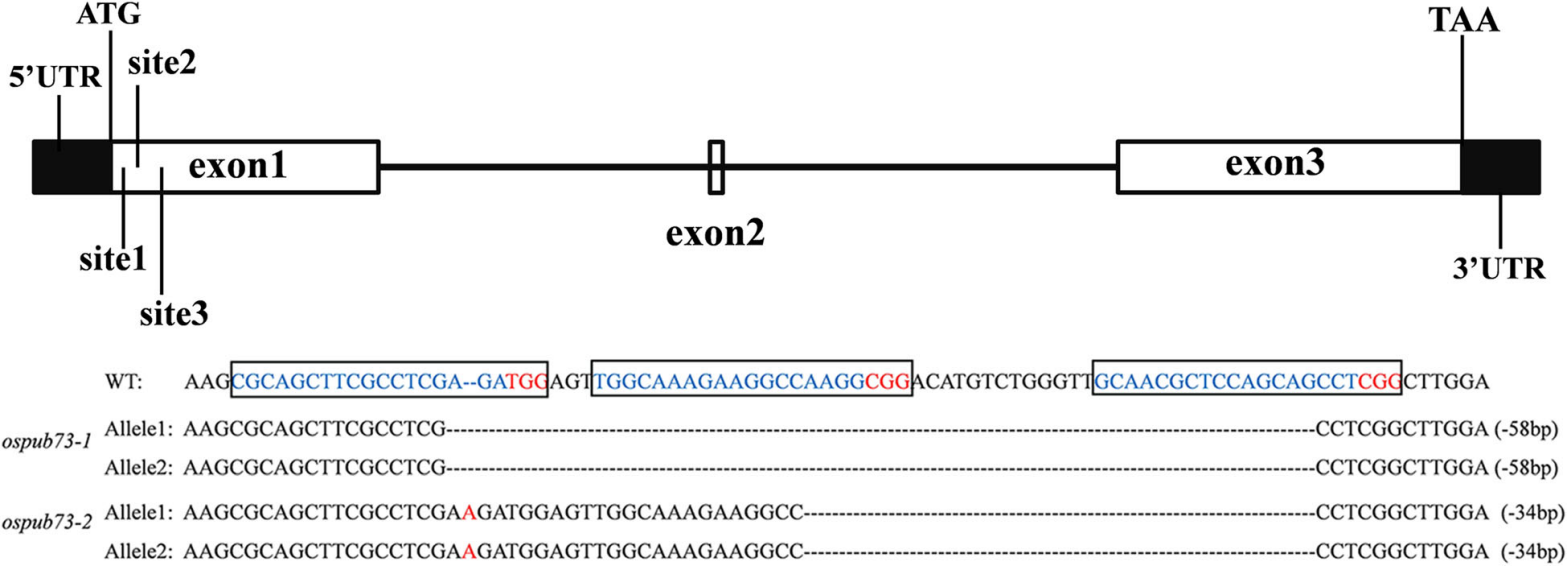
Targeted mutagenesis in rice by the CRISPR/Cas9 system. The three target sites disrupt the first exon of *ospub73*. The black boxes show target sites

To evaluate the function of *OsPUB73*, the CRISPR/Cas9 system was used to create *ospub73* mutants. The CRISPR/Cas9 recombinant vector, which included three guide RNA targets in the first exon of *OsPUB73*, was used to transform the plant of Taichung-65. A total of 18 T_0_ transgenic plants were obtained and we analyzed the target site by sequencing PCR-amplified *OsPUB73* genomic DNA from transgenic plants. There were six homozygous mutants, two bi-allelic mutants, seven heterozygous mutants, and three non-mutant plants in the transgenic plants (Additional file 4: Table S1). The *ospub73*–1 and *ospub73–2* used to subsequent experiments (Fig. 2), and the T_2_ plants of *ospub73*–1 and *ospub73–2* have been used for phenotyping and genetic analysis.

### Mutation of *OsPUB73* cause pollen semi-sterility

**Fig. 3:**
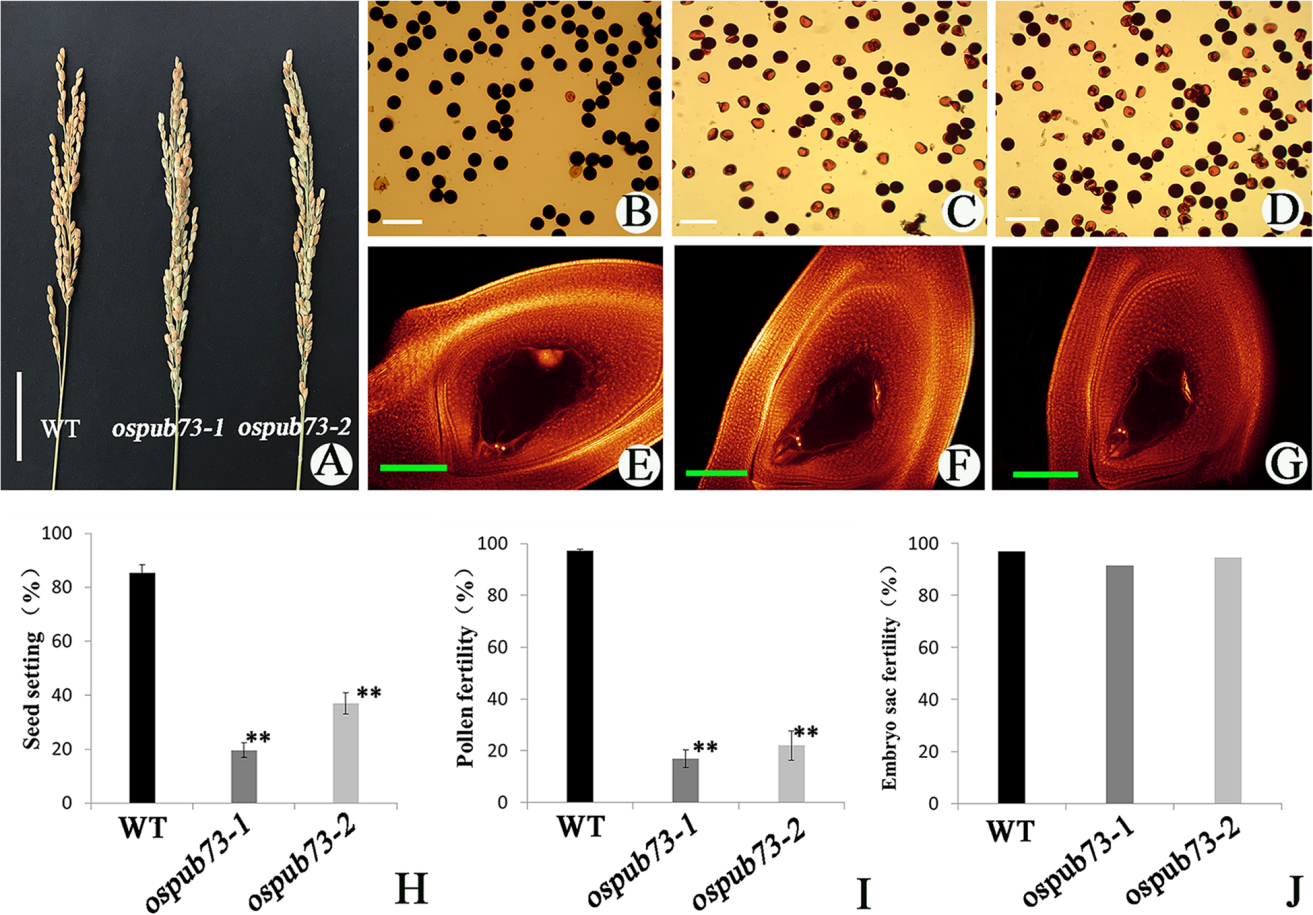
Comparison of panicle, pollen fertility, and embryo sac fertility in WT and *ospub73*. **a** panicle, bar = 5 cm; **b**-**d** pollen grains from WT (**b**), *ospub73–1* (**c**) and *ospub73–2* (**d**), bar = 100 μm; **e**-**f** embryo sac from WT (**e**), *ospub73–1*,(F) and *ospub73–2* (**g**), bar = 100 μm; **h** seed setting, sample size were *n* = 20; **i** pollen fertility, sample size were *n* = 5; **j** embryo sac fertility. ** represent *p* < 0.01. Error bars represent the SD

The *ospub73* showed normal plant type as well as normal vegetative development (Additional file 5: Figure S4). However, the panicle of the *ospub73* appeared many unfilled grains (Fig. 3a), and the seed setting of *ospub73*–1 and *ospub73–2* were only 19.63 and 37.02%, respectively, which were significantly lower than WT (85.37%) (Fig. 3h). These results implied potential defects in pollen or embryo sac development. To verify our supposition, we investigated mature embryo sac (Fig. 3e-g) and pollen fertility (Fig. 3b-d) between mutant and WT. The mature embryo sac fertility of WT and *ospub73* were nearly 90% (Fig. 3j). However, many mature pollen grains were aborted in *ospub73*, and the pollen fertility of *ospub73*–1 and *ospub73–2* were 16.85 and 22.05%, respectively, which were significantly lower than the pollen fertility of WT (Fig. 3i). These phenomena indicated that there was no difference in embryo sac fertility between WT and *ospub73*, but that *ospub73* displayed male semi-sterility. These results indicate that *OsPUB73* may be involved in the pollen development.

### Analysis of chromosome behavior and anther development in *ospub73* and WT

**Fig. 4:**
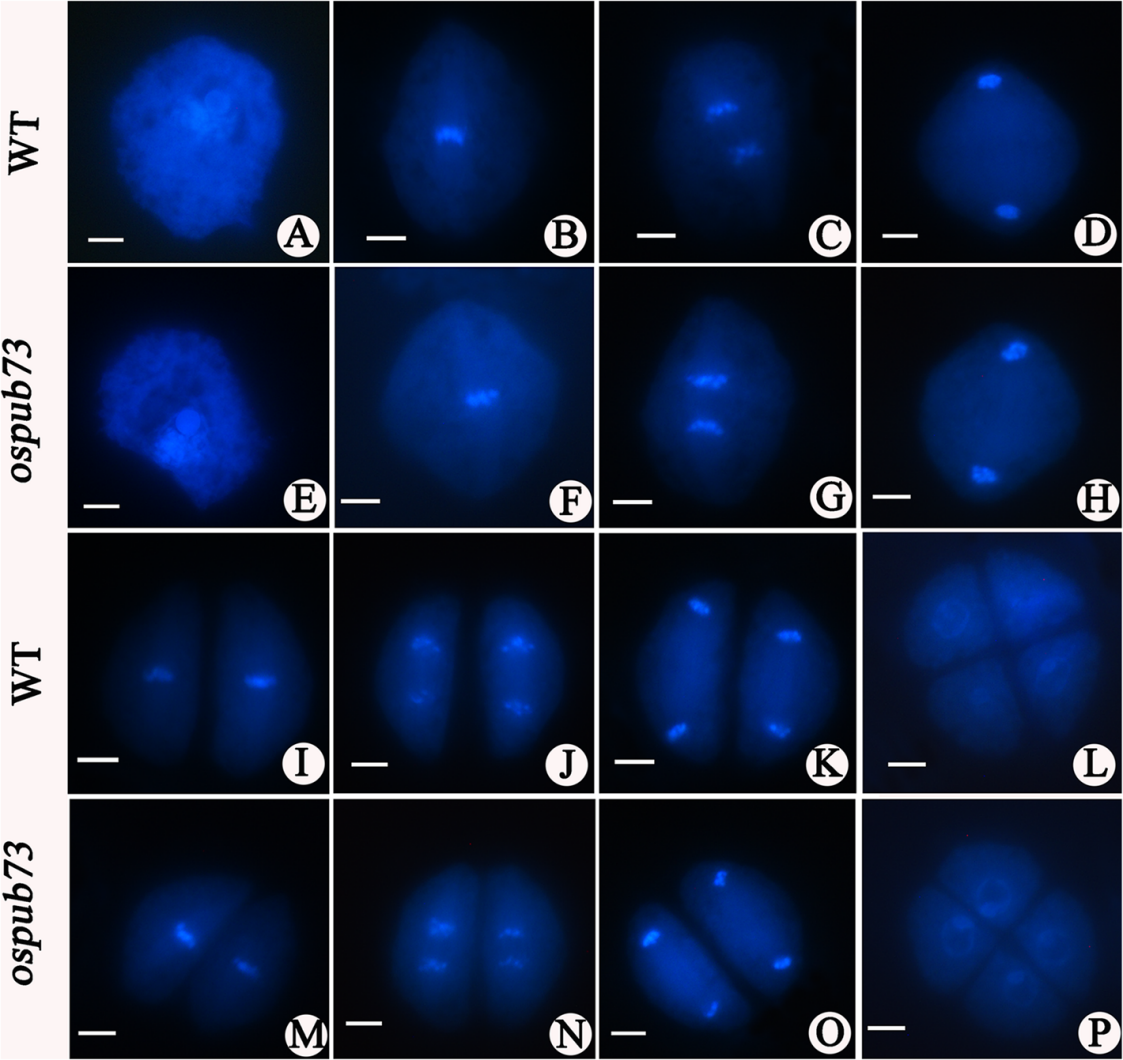
Chromosome behavior of PMC meiosis in WT and *ospub73* plant. **a** prophase I; **b** metaphase I; **c** anaphase I; **d** telophase I; **e** prophase II; **f** metaphase II; **g** anaphase II; **h** telophase II; **i** tetrad stage. Bar = 50 μm

To reveal the effects of *OsPUB73*, we compared chromosome behavior between *ospub73* and WT at pollen mother cell meiosis using DAPI (4,6–diamidino-2–phenylindole) staining. Based on the previous classification of meiotic stages [35], meiosis stages could be divided into nine development stages, including prophase I (Fig. 4a and e), metaphase I (Fig. 4b and f), anaphase I (Fig. 4c and g), telophase I (Fig. 4d and h), metaphase II (Fig. 4i and m), anaphase II (Fig. 4j and n), telophase II (Fig. 4k and o) and tetrad (Fig. 4l and p). There were no differences in chromosome behavior between *ospub73* and WT by our observation (Fig. 4).

**Fig. 5:**
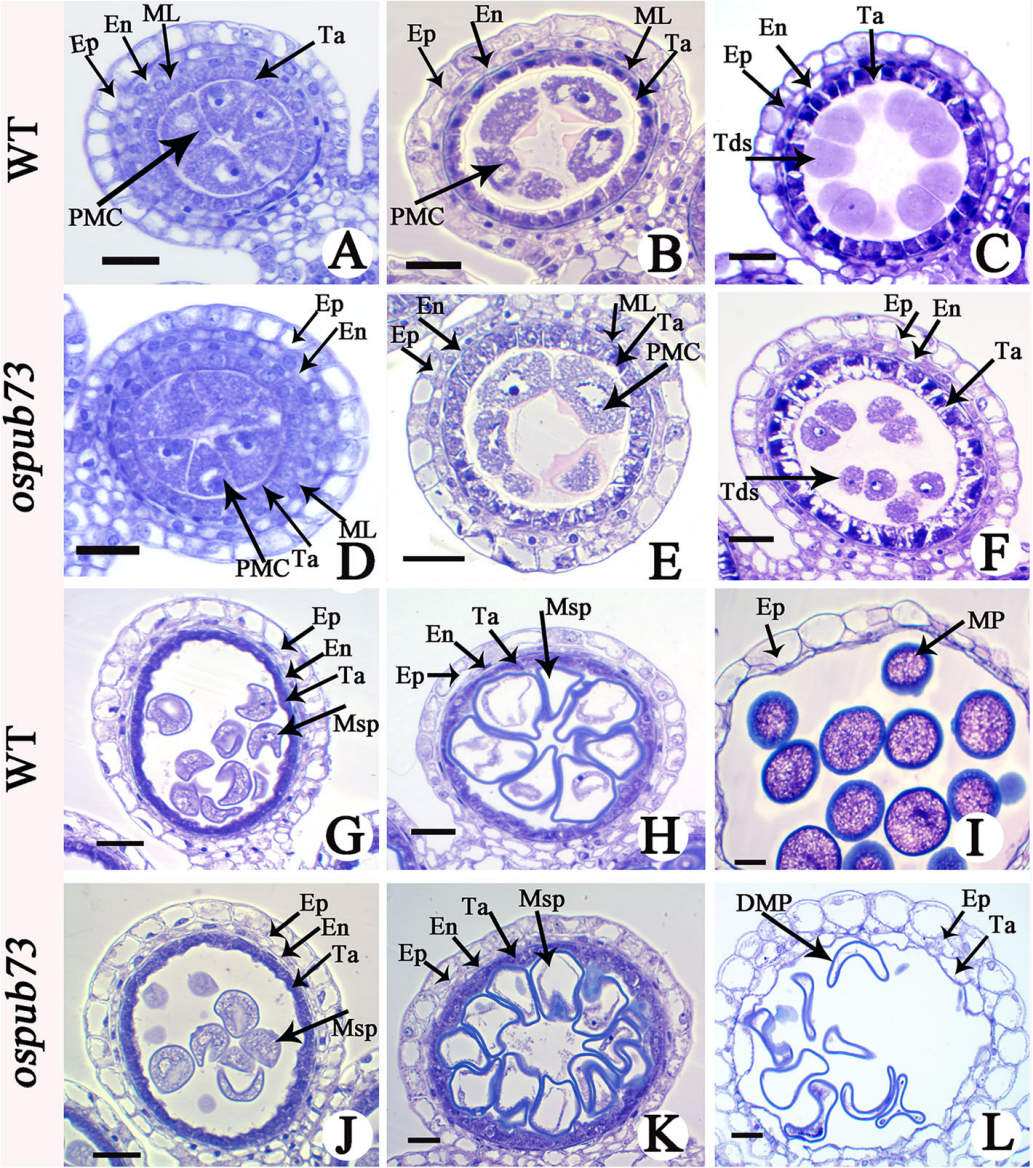
Analysis of the anther development in WT and *ospub73* plant by transverse semi-thin section. **a**–**c** and **g**–**i** showed transverse thin-section images of wild-type anther, and **d**–**f** and **j**–**l** showed transverse thin-section images of *ospub73* anther. Ep, epidermis; En, endothecium; ML, middle layer; Ta, tapetum; PMC, pollen mother cell; Tds, tetrads; Msp, microspores; MP, mature pollen; DMP, degraded mature pollen. Bars = 100 μm

**Fig. 6:**
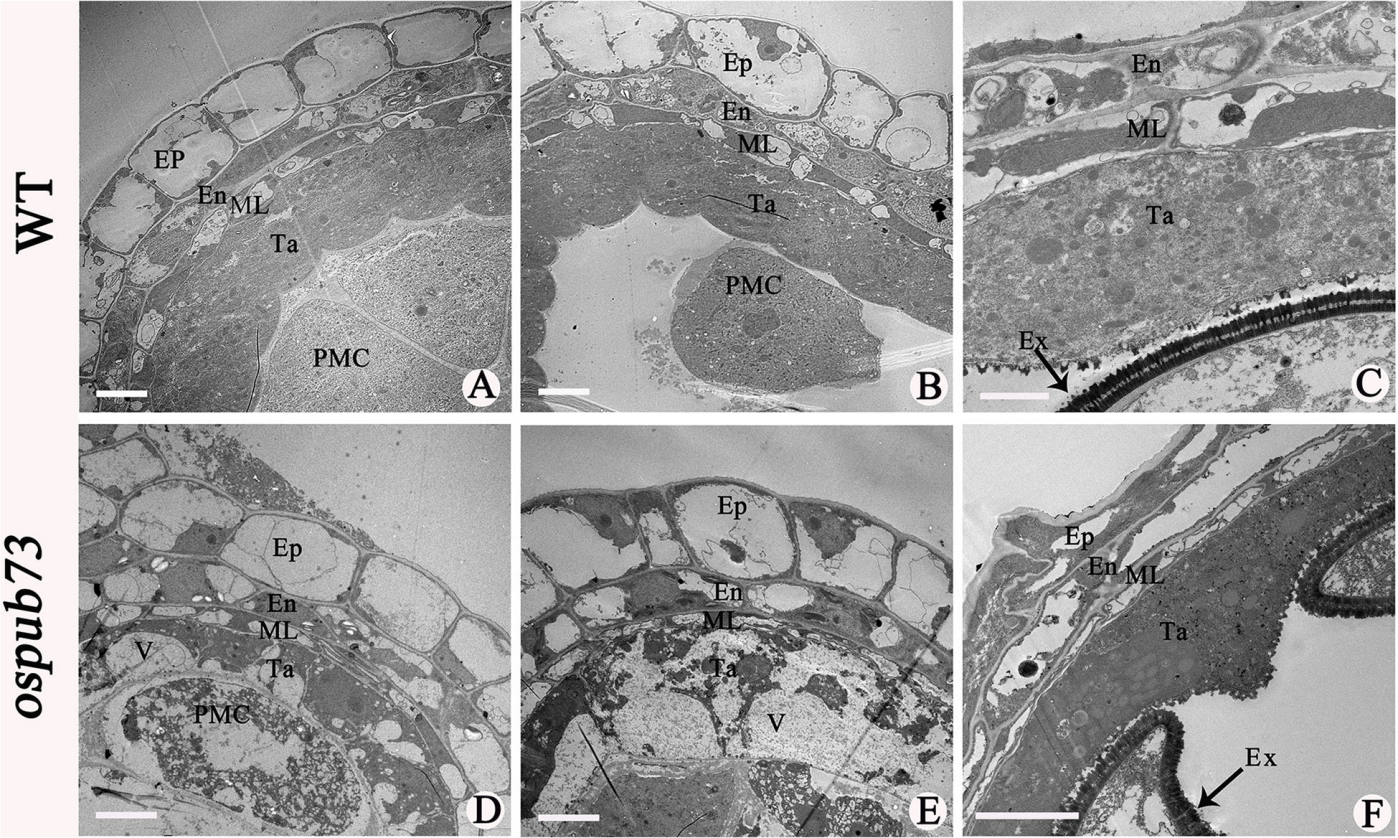
Transmission electron microscopy (TEM) analysis of anther in WT and *ospub73*. **a**, **b** and **c** present wild type anthers, (**d**), (**e**) and (**f**) presented *ospub73* anthers. Ep, epidermis; En, endothecium; ML, middle layer; Ta, tapetum; V, vacuolization; Ex, pollen exine; PMC, pollen mother cell. Bars: 5 μm in (**a**) and (**b**), (**d**) to (**f**); 2 μm in (**c**)

The above results indicated that chromosome behavior of *ospub73* is normal, which prompted us to further study *ospub73* pollen tissues. The semi-thin section analysis was performed to investigate the pollen developmental process in *ospub73* and WT. At the pre-meiosis stage, there was no obviously morphological difference between *ospub73* and WT in the anther, and the epidermis, endothecium, middle layer, tapetum and microsporocyte were normal in both *ospub73* and WT anthers (Fig. 5a and d). At meiosis stage, the pollen mother cells underwent normal meiosis and formed tetrads, and tapetum were vacuolated in *ospub73* (Fig. 5b and e). At the tetrad stage, the pollen mother cells formed tetrads and the middle layer cells became very thin and degenerated. But in *ospub73* anther, although the tetrads had formed, the tapetum seemed to be vacuolated (Fig. 5c and f). At the single microspore stage, the tapetum became more condensed and deeply stained (Fig. 5g-h and j-k). At the mature pollen stage, the WT pollen grains were full of starch and the tapetum fully degenerated. However, *ospub73* microspores were degenerated, whereas tapetum cells became more vacuolated and did not degenerate (Fig. 5i and l). In addition, the transmission electron microscopy (TEM) analysis showed the tapetum was condensed in WT (Fig. 6a and b), but the tapetum was vacuolated in *ospub73* (Fig. 6d and e). This result was consistent with semi-thin section results. Moreover, the pollen exine was abnormal at the bi-cellular pollen stage (Fig. 6f). These results suggest that *ospub73* tapetum or pollen exine exhibit abnormality in rice.

### Homozygous *ospub73* plants were identified for transcriptome analysis

In order to study the gene regulatory network that is controlled by *OsPUB73*, we analyzed the transcriptome data generated from the anthers (meiosis stage) of homozygous *ospub73–1* and WT control plants according to cytological results, which the mutant anther exhibited vacuolization during meiosis stage. The three biological replicates were established for each material. In total, about 19 million clean reads were detected in WT and *ospub73* anthers during meiosis. The clean reads were aligned against the Nipponbare reference genome, and 92.91 to 93.61% annotated transcripts of the reference genome were obtained in *ospub73* and WT rice, respectively (Table 1). The correlation coefficients were higher than 0.98 among the three biological replications (Additional file 6: Table S2), and principal component analysis (PCA) showed that replicate samples clustered together (Additional file 7: Figure S5). The correlation coefficients and PCA suggested that expression patterns have high similarity between biological replications.

**Table 1:** Overview of reads from WT and *ospub73* anthers

Sample	Total Reads	Clean reads	Mapped Reads	Unique-Mapped Reads	GC Content	Q30
WT-1	66,973,324	33,486,662	62,602,452 (93.47%)	60,953,559 (91.01%)	54.80%	93.72%
WT-2	67,440,172	33,720,086	62,884,344 (93.24%)	60,807,362 (90.16%)	54.95%	93.83%
WT-3	61,328,580	30,664,290	57,182,779 (93.24%)	55,530,879 (90.55%)	54.78%	93.53%
*ospub73–1*	65,360,720	32,680,360	60,727,192 (92.91%)	59,119,630 (90.45%)	54.45%	93.58%
*ospub73–2*	60,833,960	30,416,980	56,557,714 (92.97%)	55,052,354 (90.50%)	54.27%	93.52%
*ospub73–3*	58,140,984	29,070,492	54,423,148 (93.61%)	52,916,445 (91.01%)	53.99%	93.56%

Compared with WT anthers, a total of 2811 DEGs were found in *ospub73*, including 2240 down-regulated and 571 up-regulated genes (Additional file 8: Table S3). Gene ontology (GO) analysis showed that 85 and 4 GO were significantly enriched in the down and up-regulated DEGs, respectively. In the biological processes category, 41 GO terms were significantly enriched in the down-regulated DEGs, such as regulation of the biosynthetic process, regulation of transcription, carbohydrate metabolic process, protein amino acid phosphorylation and protein modification process; and GO term of oxidation reduction was significantly enriched in the up-regulated DEGs. In the molecular function category, 40 GO categories, such as oxidoreductase activity, protein kinase activity and kinase activity, were found to be enriched in the down-regulated DEGs while GO terms related to the oxidoreductase activity were detected in the up-regulated DEGs. In the cellular component category, a total of 4 and 2 GO terms were identified to be significantly enriched in the down and up-regulated DEGs, respectively (Additional file 9: Table S4).

**Fig. 7:**
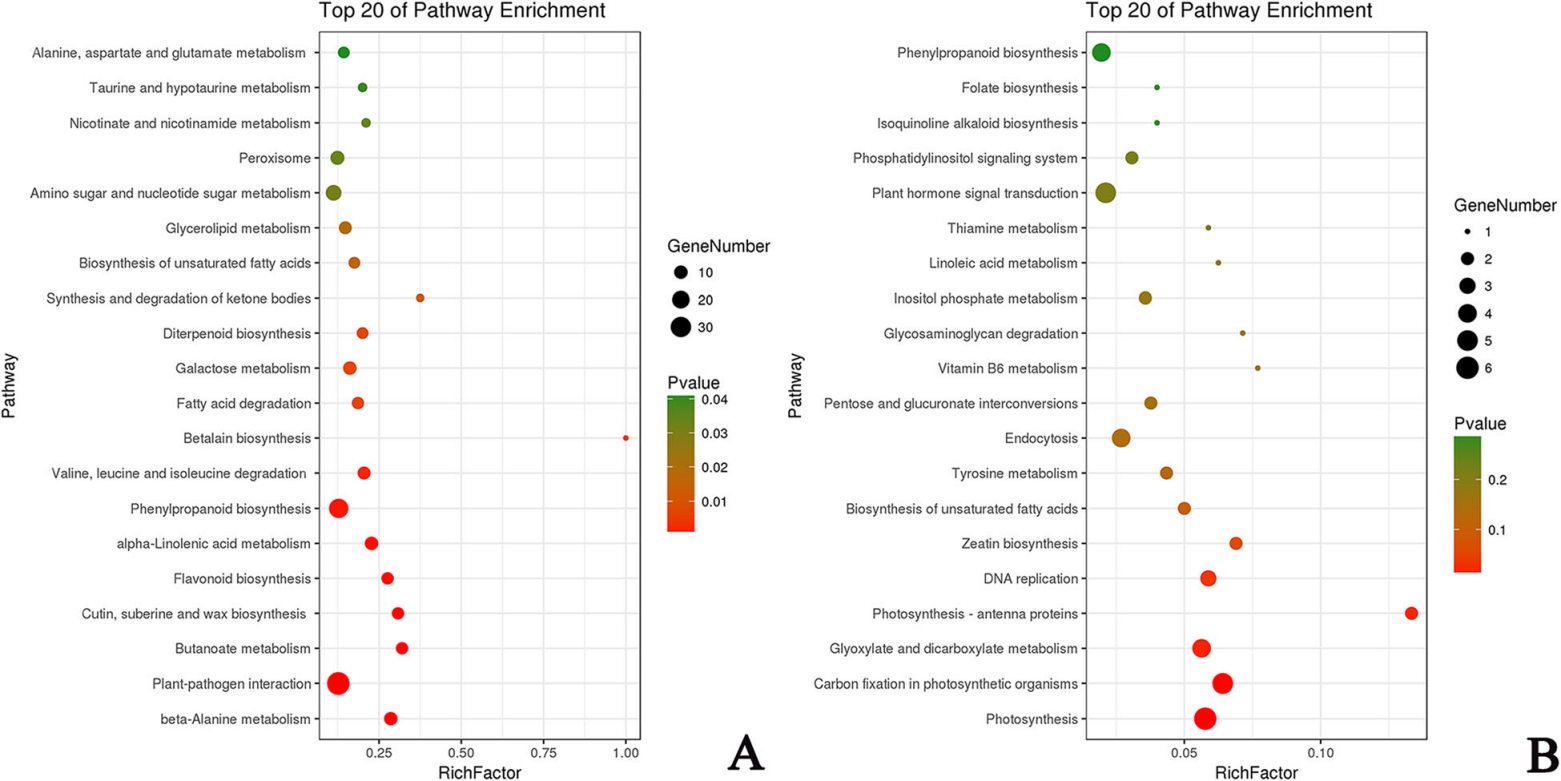
KEGG pathway assignments of DEGs. **a** KEGG analysis of down-regulated DEGs. **b** KEGG analysis of up-regulated DEGs. Both (**a**) and (**b**) show the top 20 most represented categories and the number of transcripts predicted to belong to each category

KEGG (Kyoto Encyclopedia of Genes and Genomes) analysis suggested that 109 pathways were identified in down-regulated DEGs. The top 20 most enriched pathways were Plant-pathogen interaction, Phenylpropanoid biosynthesis, Protein processing in endoplasmic reticulum, Plant hormone signal transduction, Starch and sucrose metabolism, Ubiquitin mediated proteolysis, Peroxisome in down-regulated DEGs (Fig. 7a). In total 52 pathways were identified in up-regulated DEGs. The top 20 most enriched pathways were mainly focused on the Photosynthesis, Carbon fixation in photosynthetic organisms, Plant hormone signal transduction, Glyoxylate and dicarboxylate metabolism, Endocytosis, Phenylpropanoid biosynthesis and DNA replication (Fig. 7b). The GO and KEGG analysis results showed that DEGs involved in transcription, protein modification and signal transduction were more numerous in the down-regulated DEGs.

**Fig. 8:**
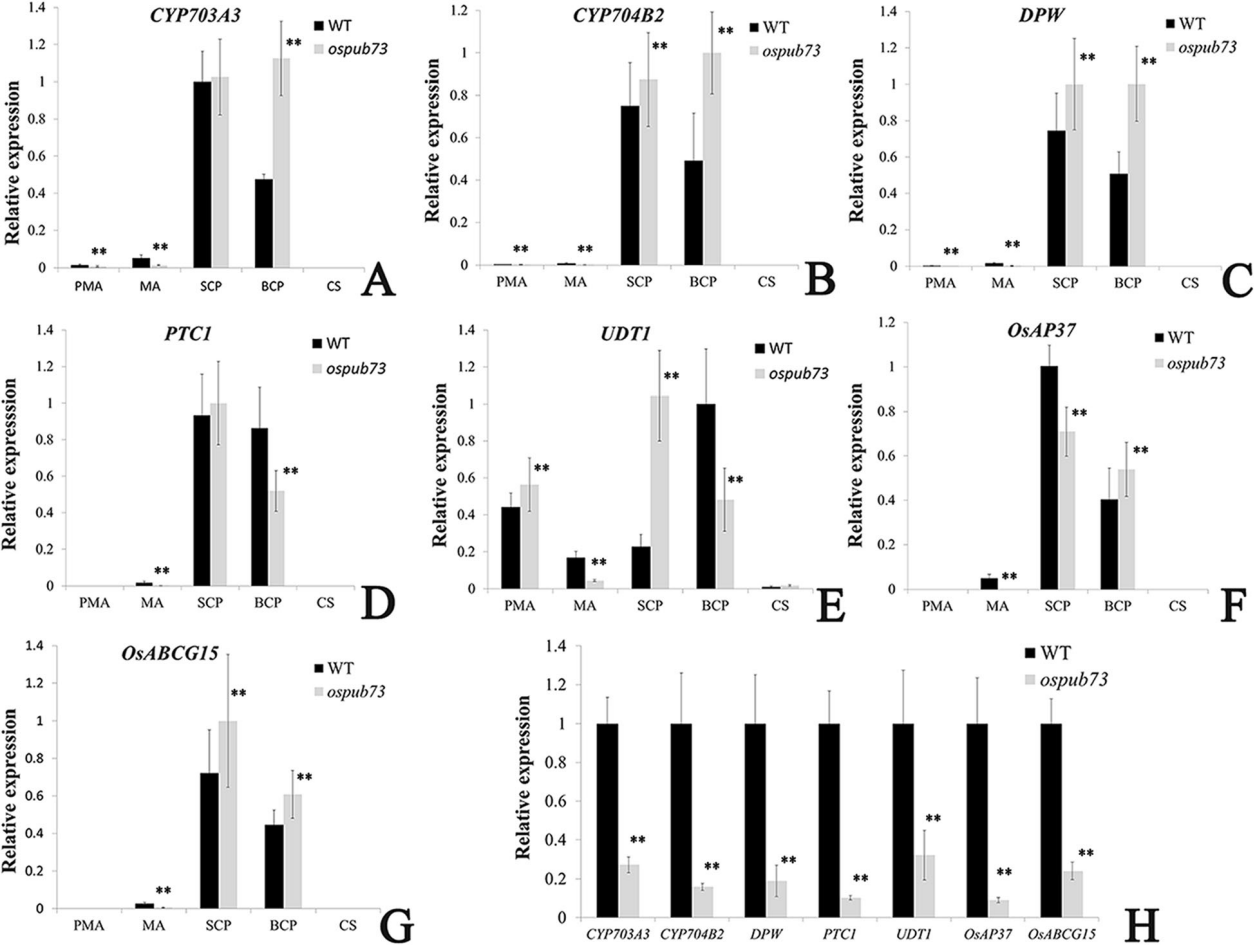
Expression analysis of genes related to anther and pollen wall synthesis in *ospub73* and WT. Expression analysis of *CYP703A3* (**a**), *CYP704B2* (**b**), *DPW* (**c**), *PTC1* (**d**), *UDT1* (**e**), *OsAP37*(F) and *OsABCG15* (**g**) in anthers during different pollen stage by normalized qRT-PCR data, and expression of seven genes in anthers at meiosis stage from the WT and *ospub73* using qRT-PCR. The X-axis represents the different stage anthers and Y-axis respresents relative expression (gene relative expression/*Actin* gene). PMA, MA, SCP, BCP and CS represent the anthers of pre-meiotic, meiosis, single microspore stage, bi-cellular pollen stage and mature pollen stage, respectively. Error bars indicate SD. ** respent *P* ≤ 0.01; Student t-test

In addition, we found that seven genes were associated with tapetum and pollen development, and these genes are down-regulated in *ospub73*, including *CYP703A3*, *CYP704B2*, *DPW*, *PTC1*, *UDT1*, *OsAP37* and *OsABCG15* (Additional file 8: Table S3)*.* The gene expression were detected in anthers from different pollen stage by using qRT-PCR (Fig. 8). The results showed that these genes were down-regulated in the *ospub73* anthers compared with WT during meiosis (Fig. 8h), and the genes expression were similar to our RNA-seq data. At the single microspore stage, the expression levels of *CYP703A3*, *CYP704B2*, *DPW*, *PTC1*, *OsAP37* and *OsABCG15* were not greatly changed between WT and *ospub73*, however, *UDT1* was up-regulated in *ospub73*. At the bi-cellular pollen stage, *CYP703A3*, *CYP704B2* and *DPW* presented high expression in the *ospub73*, and expression level of *PTC1* and *UDT1* was down-regulated in *ospub73* (Fig. 8). The *ospub73* had such a broad effects on so many important anther development genes, it is plausible to consider it as an important part of the conserved gene regulation network that regulates rice anther development.

### Comparison of *OsPUB73* regulatory role with those of *PTC1*, *UDT1*, *GAMYB* and *TDR* (*Tapetum Degeneration Retardation*) in anther development

**Fig. 9:**
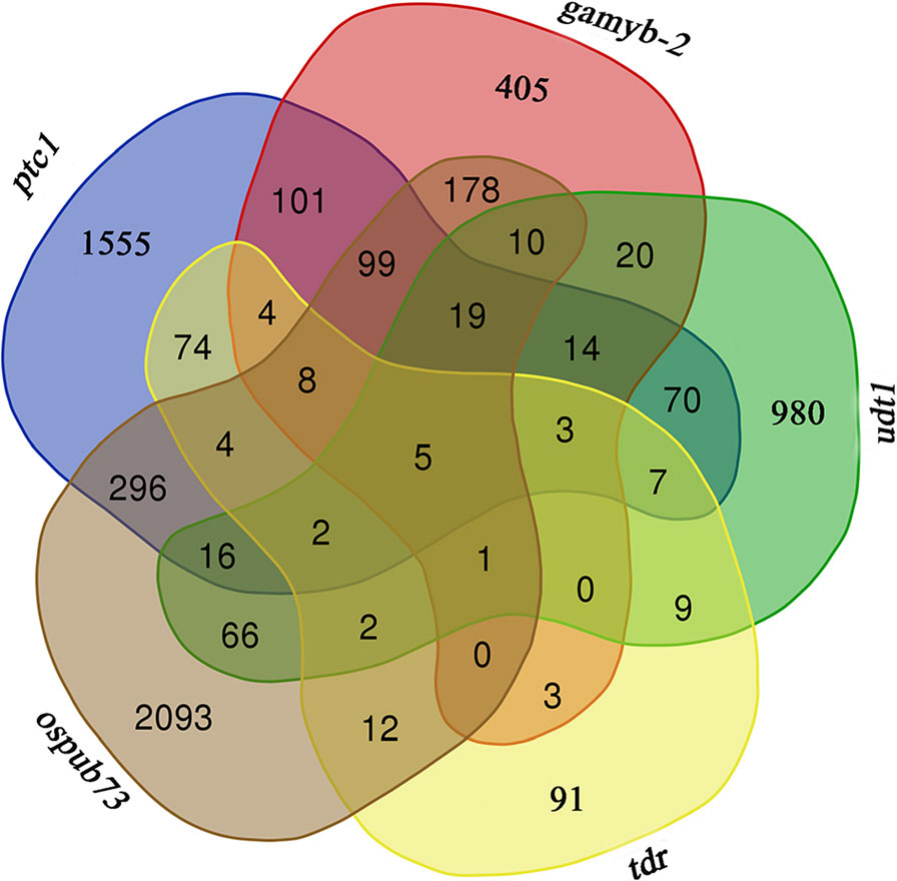
Comparison of genes expression changed in *ospub73*, *tdr*, *gamyb*, *udt1* and *ptc1* plants

To clarify characterization of *OsPUB73*, the 2811 DEGs in *ospub73* were compared with *ptc1* [17], *udt1* [13], *gamyb-2* [16] and *tdr* plants [36], and there are 18.27% (449/2458) DEGs changing in *ptc1* mutant, 9.88% (121/1255) DEGs changing in *udt1* plant, 36.78% (320/870) DEGs changing in *gamyb-2* plant and 14.72% (34/231) DEGs changing in *tdr* (Fig. 9). Five genes showed changes of expression in all five mutants (Fig. 9; Additional file 10: Table S5; Additional file 11: Figure S6), including 3-oxoacyl-reductase (*LOC_Os12g13930*), LTP (lipid transfer protein) family protein (LTPL2, *LOC_Os07g46210*), aquaporin protein (*LOC_Os01g02190*) and male sterility protein (*LOC_Os03g07140*, *DPW*) showed down-regulated expression in the all five mutants and pectinesterase (*LOC_Os07g41650*) showed down-regulated expression in *ospub73*, *udt1*, *gamyb-2* and *tdr* plants but up-regulated expression in *ptc1* (Additional file 10: Table S5). These genes are involved in lipid metabolism and transport, cell wall, and are played important roles in tapetum and pollen development.

## Discussion

### *OsPUB73* may play an essential role in male reproductive development

Many previous studies showed that the PUB possesses E3 ubiquitin ligase activity in plant, which has a significant effect on ubiquitination modification, and revealed PUB plays central roles in plant cell death, defense responses, immune reactions and flowering time [28, 29, 32, 33, 37]. In addition, a total of 77 U-box protein genes were found in rice [27]*.* The *SPL11* (*OsPUB11*) was the first PUB gene to be studied in rice, which harbored E3 ligase activity and involved in the pathway of cell death and defense [38]. Subsequently, the scientists reported *OsPUB15* [32], *OsPUB44* [39] and *OsPUB75* [40]. However, the molecular mechanisms and function PUB genes are still largely unknown. In this study, we identified a PUB gene, *OsPUB73*, which consists of three exons and two introns. The *OsPUB73* possesses E3 ligase activity in vitro [27]. We successfully developed a homozygous *ospub73* by CRISPR/Cas9 system. The *ospub73* showed normal embryo sac fertility. However, *ospub73* showed male semi-sterility.

Meiosis chromosome behavior and anther wall development are essential parts of the correlated plant male reproductive development [41, 42]. The tapetum supplies nutrients and stable environment for microspore development, and the timely degradation of tapetum is crucial for pollen grain formation [4, 43]. The PUB genes were also discovered to have important function in tapetum development and thus affected male reproductive development. Wang et al. [44] reported a PUB gene (*AtPUB4*) taking part in pollen development in *Arabidopsis*. The *Atpub4* had abnormal expansion of the tapetum layer after the tetrad stage and tapetum layer incomplete degeneration in the end, and absence of *AtPUB4* leads to complete male sterility, these results suggested *AtPUB4* may be a crucial factor in male sterility. We observed no differences in chromosome behavior between *ospub73* and WT by using DAPI staining observation, but the tapetum layer didn’t degenerate during mature pollen stage and generated aborted pollen in *ospub73*. These observations suggested knock-out of *OsPUB73* may cause semi-sterility in rice.

### *OsPUB73* may affect the regulatory network of the genes associated with tapetum or pollen exine development in rice

It is well known that plant male reproductive development is a complex biological process and a large number of genes take part in this process [45, 46]. Recently, RNA sequencing (RNA-seq) has been found to be a helpful tool for investigating gene expression and researching gene regulated expression networks [47, 48]. The RNA-seq analysis showed 79.69% DEGs were down-regulated in the *ospub73* compared with WT. This indicated that down-regulated genes may play important roles in male reproduction. Among the down-regulated genes, many DEGs were enriched in the carbohydrate metabolic process, lipid metabolic process and protein modification process, which are related to anther development or pollen wall generation.

Furthermore, we identified seven down-regulated genes in *ospub73*, which were associated with tapetum development, including *CYP703A3*, *CYP704B2*, *DPW*, *PTC1*, *UDT1*, *OsAP37* and *OsABCG15*. *UDT1* encodes a helix-loop-helix protein, which is required for tapetal degradation in rice. In the *udt1* mutant, the tapetum becomes vacuolated at the meiosis stage and there is no pollen in anther locules [13]. *CYP703A3* and *CYP704B2* are cytochrome P450s family genes in rice, which have been specifically detected in the anther and catalyzed hydroxylation of fatty acids. Loss of function of *CYP703A3* and *CYP704B2* genes caused defective pollen exine and male reproductive development, and the *CYP703A3* was directly regulated by *TDR* [9, 12, 49]. *DPW* is a fatty acyl carrier protein reductase and is required for the anther cuticle development and pollen sporopollenin biosynthesis, and the *dpw* mutant exhibits abnormalities of anther surface and pollen wall and reduction of lipidic Ubisch bodies [10]. *OsAP37* is an aspartic protease that is directly regulated by *EAT1* (*ETERNAL TAPETUM 1*) and caused abnormal tapetum in rice [50]. *PTC1*, which is a PHD-finger protein, controls tapetal PCD in the rice anther development, and absence of *PTC1* resulted in changed pollen wall structure and male sterility [17]. *OsABCG15* encodes an ATP binding cassette transport protein that plays an important role in pollen exine development by exporting lipids from tapetem to anther locules [51, 52]. The above genes are essential for tapetum or pollen development. The abrupt alteration of the expression patterns of these tapetum-related genes may cause abnormal tapetum and lead to male semi-sterility in *ospub73*.

Four transcription factors have been identified to play essential roles in tapetum formation and degeneration in rice, including *GAMYB*, *UDT1*, *TDR* and *PTC1*. *GAMYB* is a MYB family transcription factor, *UDT1* and *TDR* encode bHLH family transcription factor, and *PTC1* is a PHD-finger transcription factor. The four mutants showed delayed tapetum degeneration and their pollen exine was defective [13, 15–17, 36]. The *gamyb*, *udt1*, *tdr* and *ptc1* mutants showed the same phenotype during pollen development, including delayed tapetum degeneration and microspore abortion. In *ospub73*, we also observed the phenomenon of delayed degradation of tapetum. Furthermore, we found no changes in the expression of *OsPUB73* in the *gamyb*, *udt1*, *tdr* and *ptc1* plants. In addition, we compared the regulatory networks of *OsPUB73*, *GAMYB*, *UDT1*, *TDR* and *PTC1* [13, 16, 17, 36], and observed that five key genes are co-regulated by *OsPUB73*, *GAMYB*, *UDT1*, *TDR* and *PTC1*, including *DPW*, LTP precursor (*LOC_Os07g46210*), aquaporin protein (*LOC_Os01g02190*) and pectinesterase (*LOC_Os07g41650*). Interestingly, these five genes were almost down-regulated in the five mutants except for *LOC_Os07g41650* being up-regulated in *ptc1*. These five genes regulate metabolism and transport of metabolites involved in tapetum or pollen wall development. For example, *DPW* is a putative fatty acid reductase and plays important roles in pollen wall development [10]. *DPW* is down-regulated in all five mutants. As reported, LTPs are related to transport lipidic component from tapetum to the microspore in anther, and are crucial for rice pollen wall formation [4, 11]. *LOC_Os07g46210* belongs to LTP family in rice, and it is down-regulated in all five mutants. These results showed that these five genes play essential roles in all five mutants and may be important factors in tapetum development.

## Conclusions

In this study, we obtained *ospub73* homozygous mutant on a *japonica* rice variety (Taichung65) by CRISPR/Cas9 system. The *ospub73* showed normal vegetative development and mature embryo sac fertility, but exhibited semi-sterility of pollen grain. The cytological observation showed that *ospub73* tapetum vacuolated during meiosis stage, and pollen exine exhibited abnormal phenomenon at the bi-cellular pollen stage. In addition, some important tapetum-related genes are down-regulated in *ospub73* compared with WT. We speculated that the relationships of these genes are not a simple linear regulatory gene network but there is instead a complex gene regulatory network in male reproductive development. This work provides new insights into the role of PUB in rice male reproductive development.

## Methods

### Materials

The *japonica* cultivar Taichung-65 was used as WT. Taichung-65 plants were planted at the experimental farm of South China Agricultural University (SCAU) under natural conditions.

### Development and identification of mutant rice

*OsPUB73* mutants were generated using the CRISPR/Cas9 system as previously reported [53]. The three target site sequences of *OsPUB73* were cloned into the single guide RNA (sgRNA), and the integrated sgRNA expression cassettes of *OsPUB73* were incorporated into the CRISPR/Cas9 vector pLYCRISPR/Cas9Pubi-H. Then, the vectors were transferred into Taichung-65. The genomic DNAs of transgenic lines and WT were extracted from young leaves using the CTAB method [54]. The genomic region surrounding the CRISPR target site for *OsPUB73* was amplified by PCR, and the segment was subjected to Sanger sequencing to screen for mutants. The T_2_ plants of homozygous mutant have been used for phenotyping and genetic analysis. The primer sequences used in this study are listed in Additional file 12: Table S6.

### Observation of chromosome behavior

The spikelet was collected from *ospub73* and WT with −2 to 2 cm between their flag leaf cushion and the second to last leaf cushion, and fixed in Carnoy solution (ethanol: acetic acid = 3:1) over 24 h. Then the samples were stored in 70% ethanol at 4 °C after washing two times with 70% ethanol at 20 min. Anthers were dissected from the floret and placed in a small drop of 1 mg/L DAPI on a glass slide. After 5–10 min, the glass slide was covered with a slide cover and was observed under a fluorescence microscope (Leica DMRXA).

### Characterization of *ospub73* phenotype

The whole mount eosin B confocal laser scanning microscopy (WE-CLSM) was used to investigate the embryo sac fertility in *ospub73* and WT according to Chen et al. [55] with minor modifications. The mature spikelet was collected and fixed in FAA (50% ethanol: acetic acid: methanol = 89:6:5). The ovary was removed from the inflorescences, and was rehydrated, stained for eosin B, dehydrated and shifted into a mixed solution (ethanol and methyl salicylate = 1:1). Finally, the ovary was placed in pure methyl salicylate and examined with a laser scanning confocal microscope (Leica SPE). The pollen fertility of *ospub73* and WT were observed according to Chen et al. [35]. For the semi-thin assay, the anthers of *ospub73* and WT control plants at different pollen developmental stages were collected and fixed in FAA over 48 h at room temperature. After dehydration through an ethanol series, tissues were embedded in a Leica 7022 Histeresin Embedding Kit (7022LR) according to the manufacturers’ protocol (Heraeus Kulzer). Sections of 2 to 3 μm thickness were cut with the microtome (Leica RM2235) and were dried at 60 °C for 24 h. The sections were stained in 0.5% toluidine blue (m/v). The sections were observed and photographed under a microscope (Motic BA200). For the TEM assay, the anthers were collected for fixation, and the process was performed as according to Li et al. [56].

### Real-time quantitative polymerase chain reaction (qRT-PCR) assay

Total RNA was isolated from frozen samples using TRIzol reagent (Invitrogen, USA) according to the manufacturer’s instructions. The first-strand cDNA was synthesized using a Prime Script RT reagent Kit with gDNA Eraser (TaKaRa) (Code No.RR047A, TaKaRa) according to the manufacturer’s instructions. The qRT-PCR reaction was performed on the Roche LightCycler480 by using the TB Green Premix Ex Taq II (Code No.RR820A, TaKaRa), and qRT-PCR reaction process was performed according to Chen et al. [35]. All qRT-PCR reactions were performed in three biological replicates. The primers for qRT-PCR are shown in Additional file 12: Table S6.

### RNA-seq experiments and data analysis

The anthers of T_2_ transgenic lines (homozygous mutant) and WT control plants at the meiotic stage were collected in three biological replicates at −80 °C for RNA isolation. Total RNA was taken according to the manual instructions of the TRIzol Reagent (Life technologies, California, USA). The RNA-seq process was performed according to a previously described approach [35]. The gene expression differences between samples were detected using the DESeq package. The DEGs were identified with FDR (false discovery rate) ≤ 0.01 and the absolute value of log2 (Fold change) ≥1, and then DEGs were used for subsequent analysis.

## Supplementary information

**Additional file 1: Figure S1.** Amplification of *OsPUB73* CDS.

**Additional file 2: Figure S2.** Amino acid sequence alignment of *OsPUB73* with other V class genes in rice and *Arabidopsis*.

**Additional file 3: Figure S3.** The expression pattern analysis of *OsPUB73* in Taichung 65.

**Additional file 4: Table S1.** List of the T_0_ result of knockout *OsPUB73* by CRISPR/Cas9.

**Additional file 5: Figure S4.** Plant phenotype of *ospub73* and WT plants. (PPTX 399 kb)

**Additional file 6: Table S2.** The correlation analysis between all samples.

**Additional file 7: Figure S5.** The principal component analysis (PCA) in WT and mutant plant.

**Additional file 8: Table S3.** Differentially expressed genes between WT and mutant.

**Additional file 9: Table S4.** Gene ontology (GO) enrichment analysis for differently expressed genes between WT and mutant plant.

**Additional file 10: Table S5.** Changed expression of genes in *ospub73*, *ptc1*, *tdr*, *gamyb-2* and *udt1*.

**Additional file 11: Figure S6.** The five important genes expression were confirmed by qRT-PCR in *ospub73* and WT.

**Additional file 12: Table S6.** The primers were used in this study.

## Data Availability

The RNA-seq data are available from the NCBI under the accession number PRJNA578476. All data supporting the conclusions described here are provided in tables, figures, and additional files.

## Abbreviations

DAPI: 4,6–diamidino-2–phenylindole
DEGs: Differentially expressed genes
FDR: False discovery rate
GO: Gene ontology
KEGG: Kyoto Encyclopedia of Genes and Genomes
LTP: Lipid transfer protein
PCA: Principal component analysis
PCD: Programmed cell death
PUB: Plant U-box
qRT-PCR: Quantitative real-time polymerase chain reaction
RT-PCR: Rverse transcription polymerase chain reaction
TEM: Transmission electron microscopy
WT: Wild type
