# Supplementary figures and images for: Cytological and transcriptome analyses reveal OsPUB73 defect affects the gene expression associated with tapetum or pollen exine abnormality in rice
Source: BMC Plant Biol. 2019 Dec 10;19:546. doi: 10.1186/s12870-019-2175-2 (PMC6902612; doi:10.1186/s12870-019-2175-2)

## Slide 1
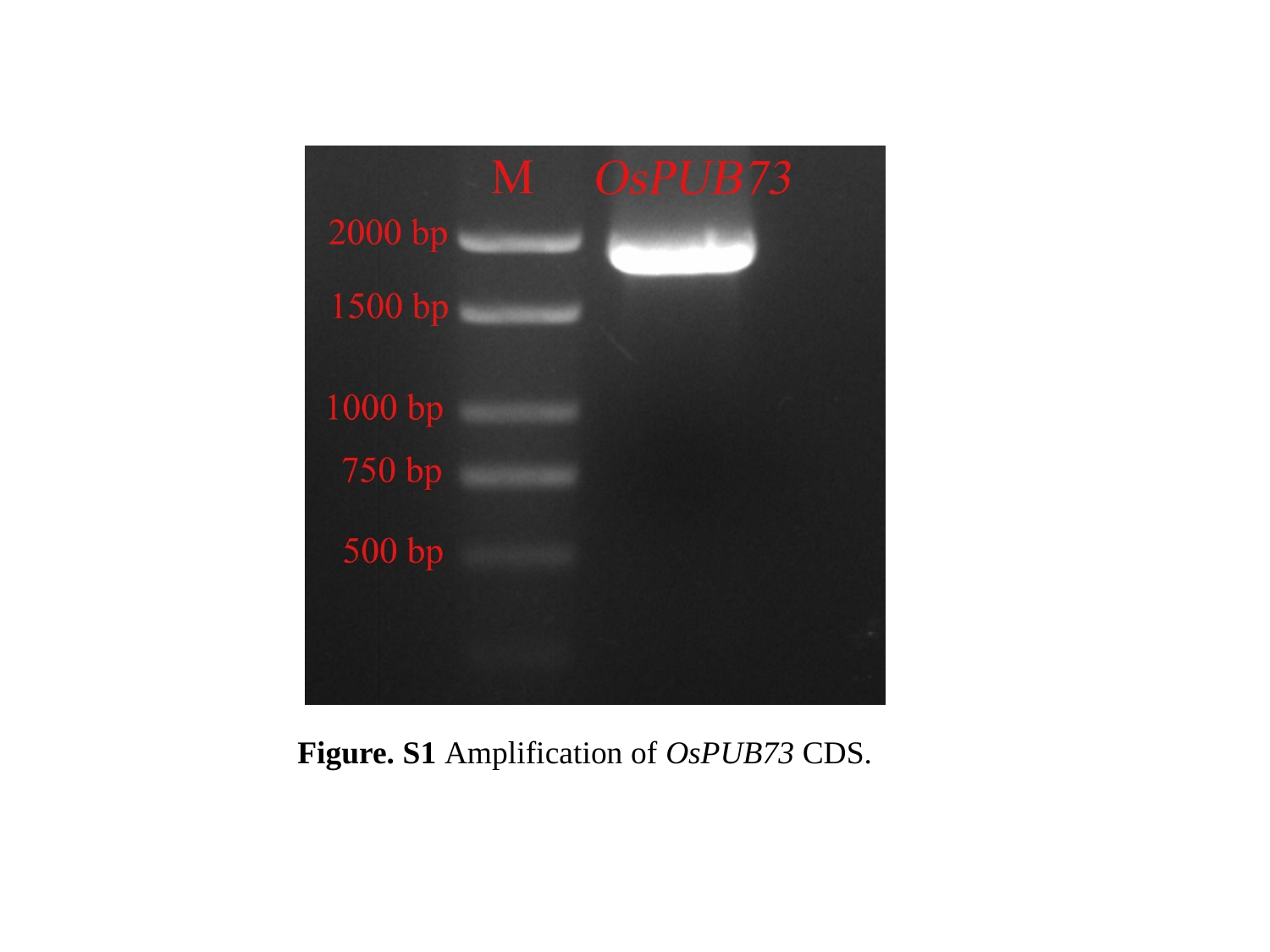

Figure. S1 Amplification of OsPUB73 CDS.

Supplement: Supplementary file 1 — Additional file 1: Figure S1. Amplification of OsPUB73 CDS. [file 12870_2019_2175_MOESM1_ESM.pptx]

## Slide 1
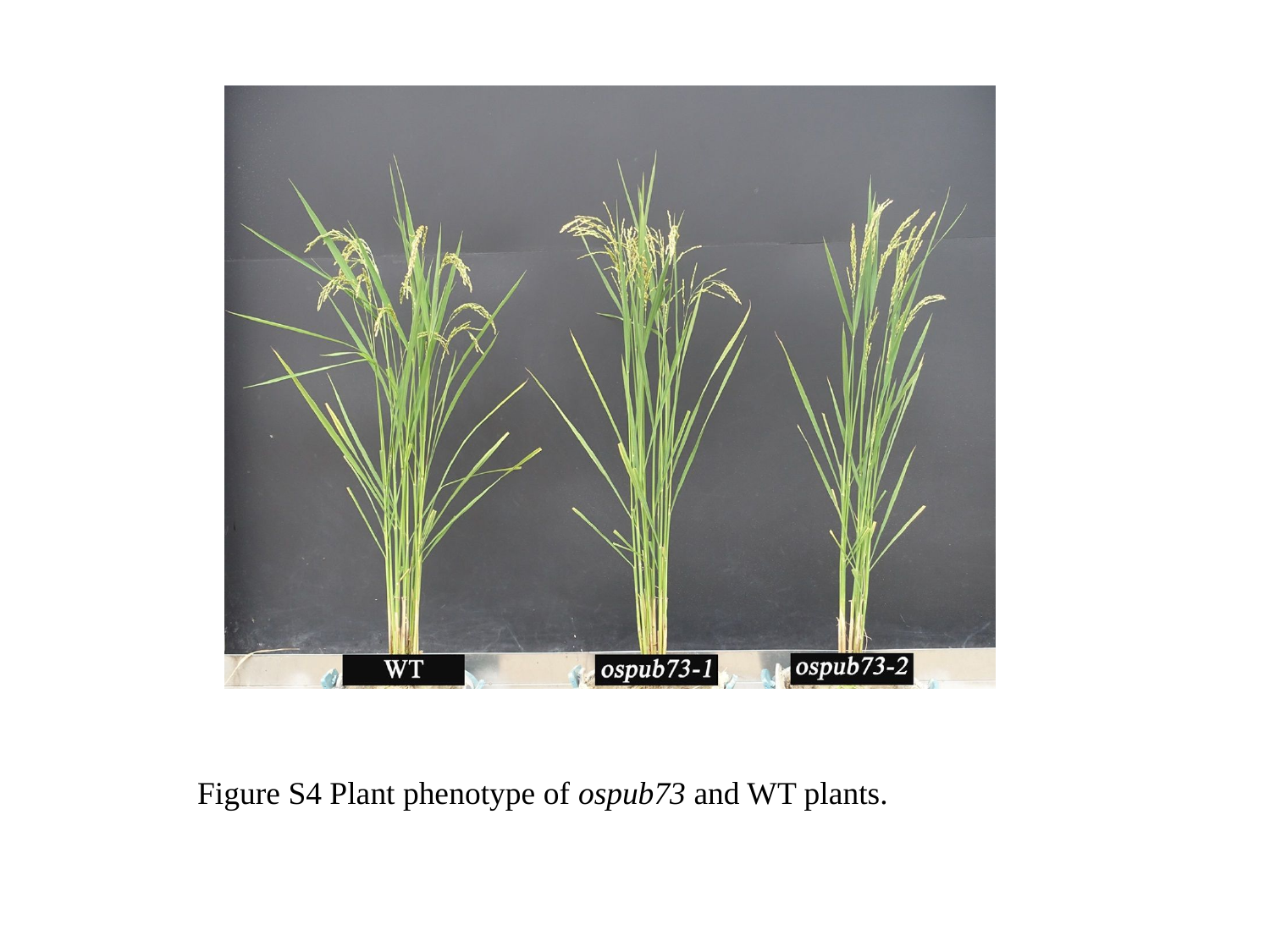

Figure S4 Plant phenotype of ospub73 and WT plants.

Supplement: Supplementary file 5 — Additional file 5: Figure S4. Plant phenotype of ospub73 and WT plants. (PPTX 399 kb) [file 12870_2019_2175_MOESM5_ESM.pptx]

## Slide 1
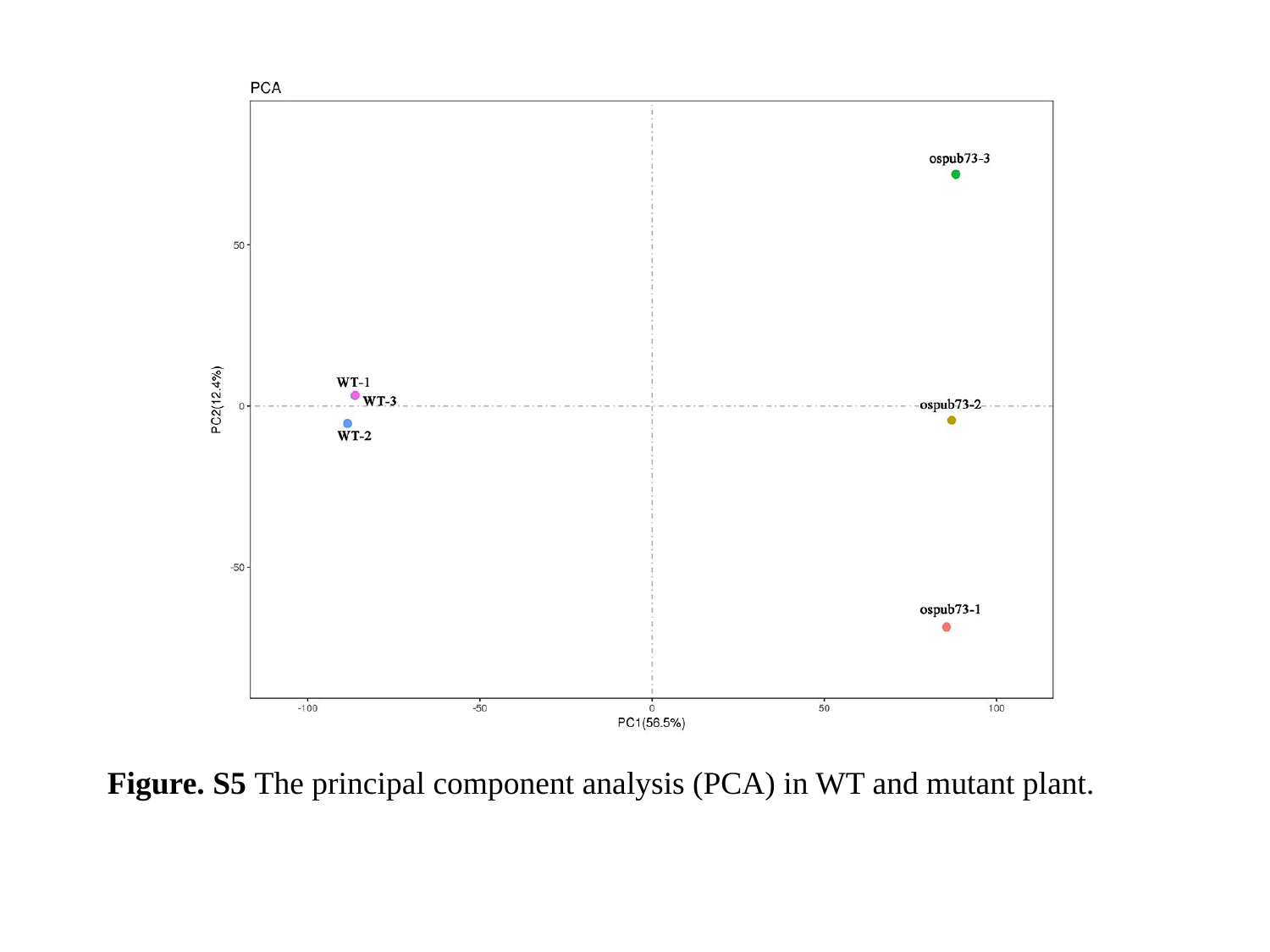

Figure. S5 The principal component analysis (PCA) in WT and mutant plant.

Supplement: Supplementary file 7 — Additional file 7: Figure S5. The principal component analysis (PCA) in WT and mutant plant. [file 12870_2019_2175_MOESM7_ESM.pptx]

## Slide 1
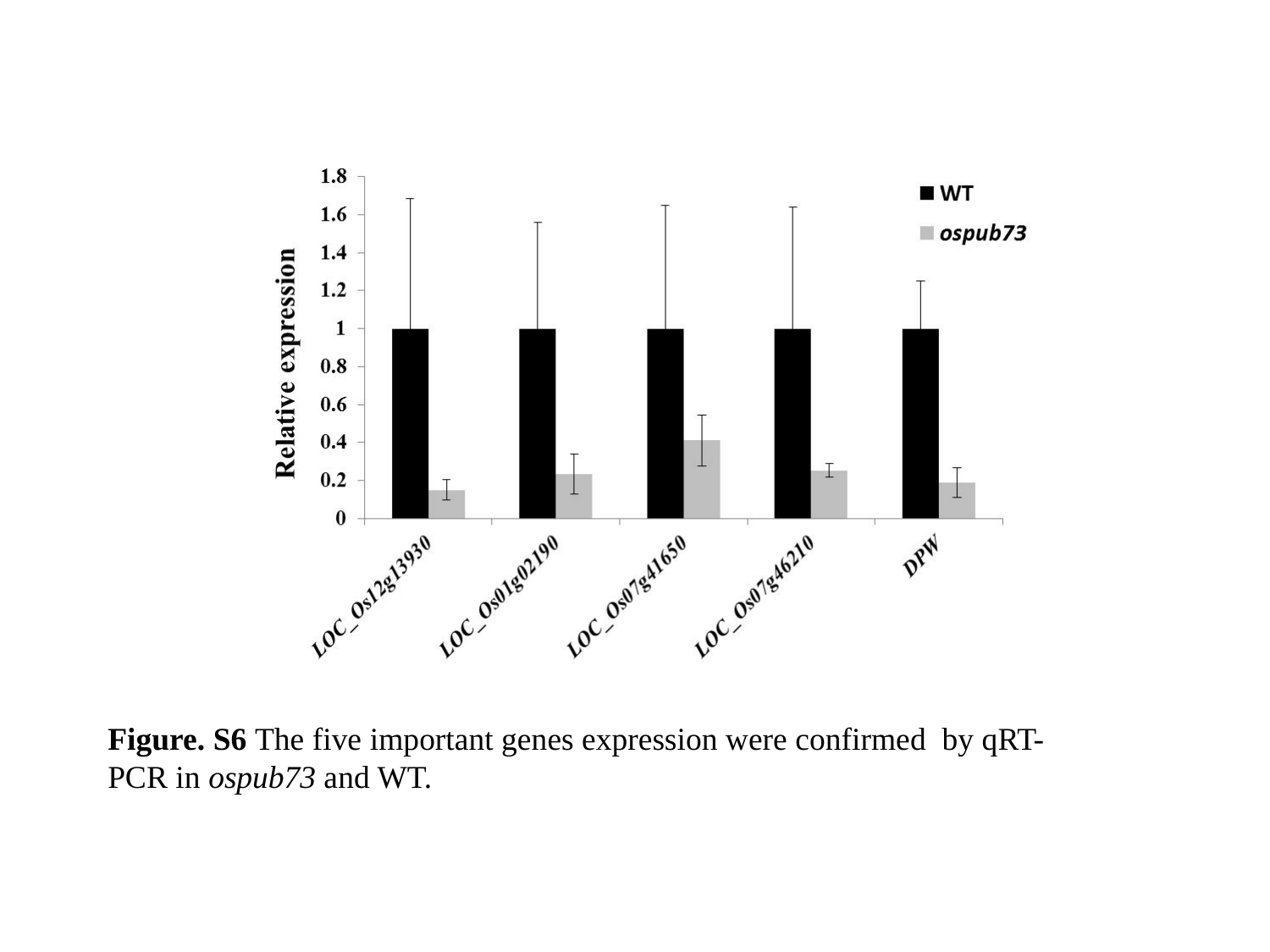

Figure. S6 The five important genes expression were confirmed by qRT-PCR in ospub73 and WT.

Supplement: Supplementary file 11 — Additional file 11: Figure S6. The five important genes expression were confirmed by qRT-PCR in ospub73 and WT. [file 12870_2019_2175_MOESM11_ESM.pptx]
