# Supplementary material for: Cytological and transcriptome analyses reveal OsPUB73 defect affects the gene expression associated with tapetum or pollen exine abnormality in rice
Source: BMC Plant Biol. 2019 Dec 10;19:546. doi: 10.1186/s12870-019-2175-2 (PMC6902612; doi:10.1186/s12870-019-2175-2)
Supplement: Supplementary file 2 — Additional file 2: Figure S2. Amino acid sequence alignment of OsPUB73 with other V class genes in rice and Arabidopsis. [file 12870_2019_2175_MOESM2_ESM.pptx]

## Slide 1
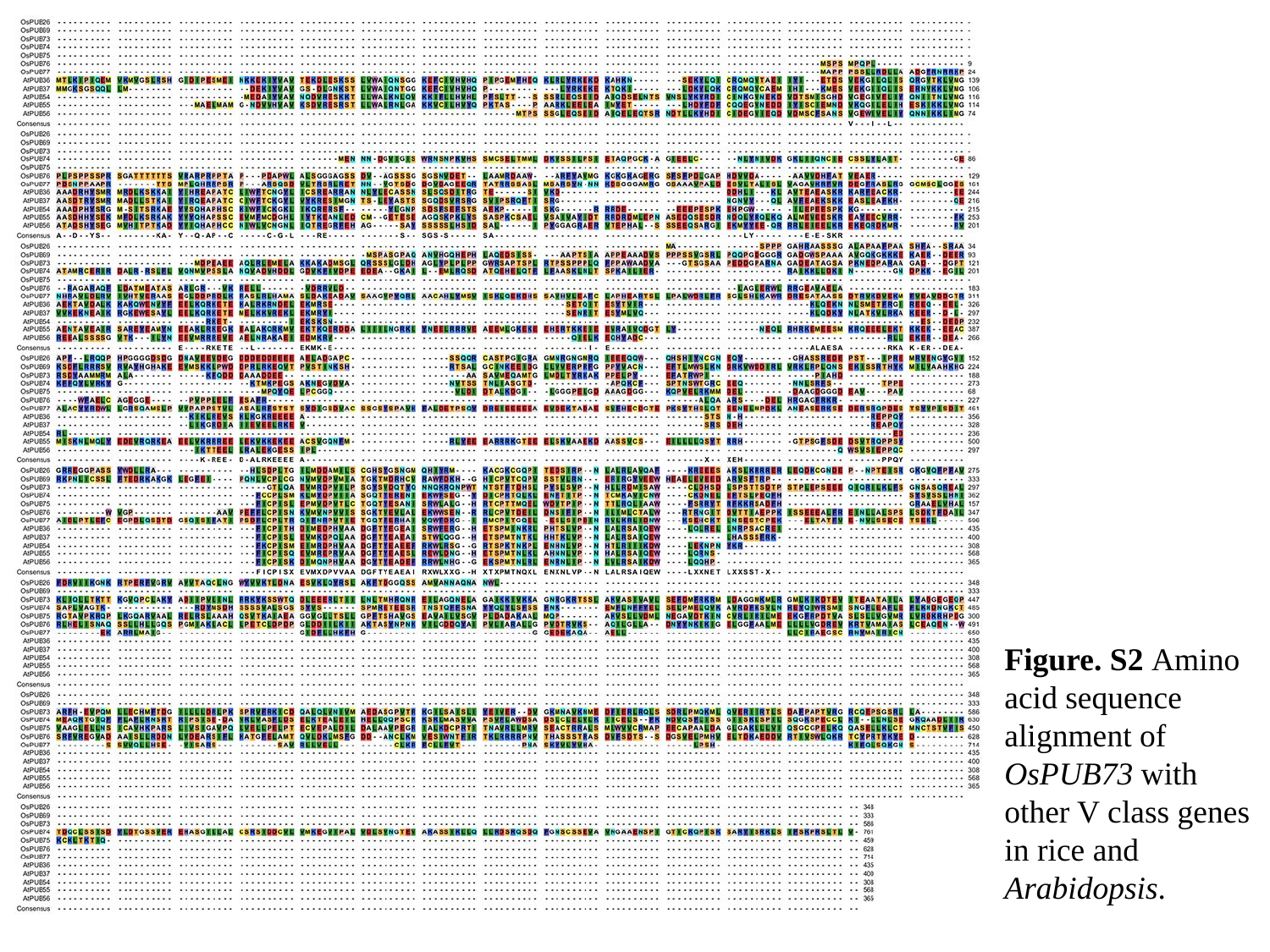

Figure. S2 Amino acid sequence alignment of OsPUB73 with other V class genes in rice and Arabidopsis.
