# Supplementary material for: Cytological and transcriptome analyses reveal OsPUB73 defect affects the gene expression associated with tapetum or pollen exine abnormality in rice
Source: BMC Plant Biol. 2019 Dec 10;19:546. doi: 10.1186/s12870-019-2175-2 (PMC6902612; doi:10.1186/s12870-019-2175-2)
Supplement: Supplementary file 3 — Additional file 3: Figure S3. The expression pattern analysis of OsPUB73 in Taichung 65. [file 12870_2019_2175_MOESM3_ESM.pptx]

## Slide 1
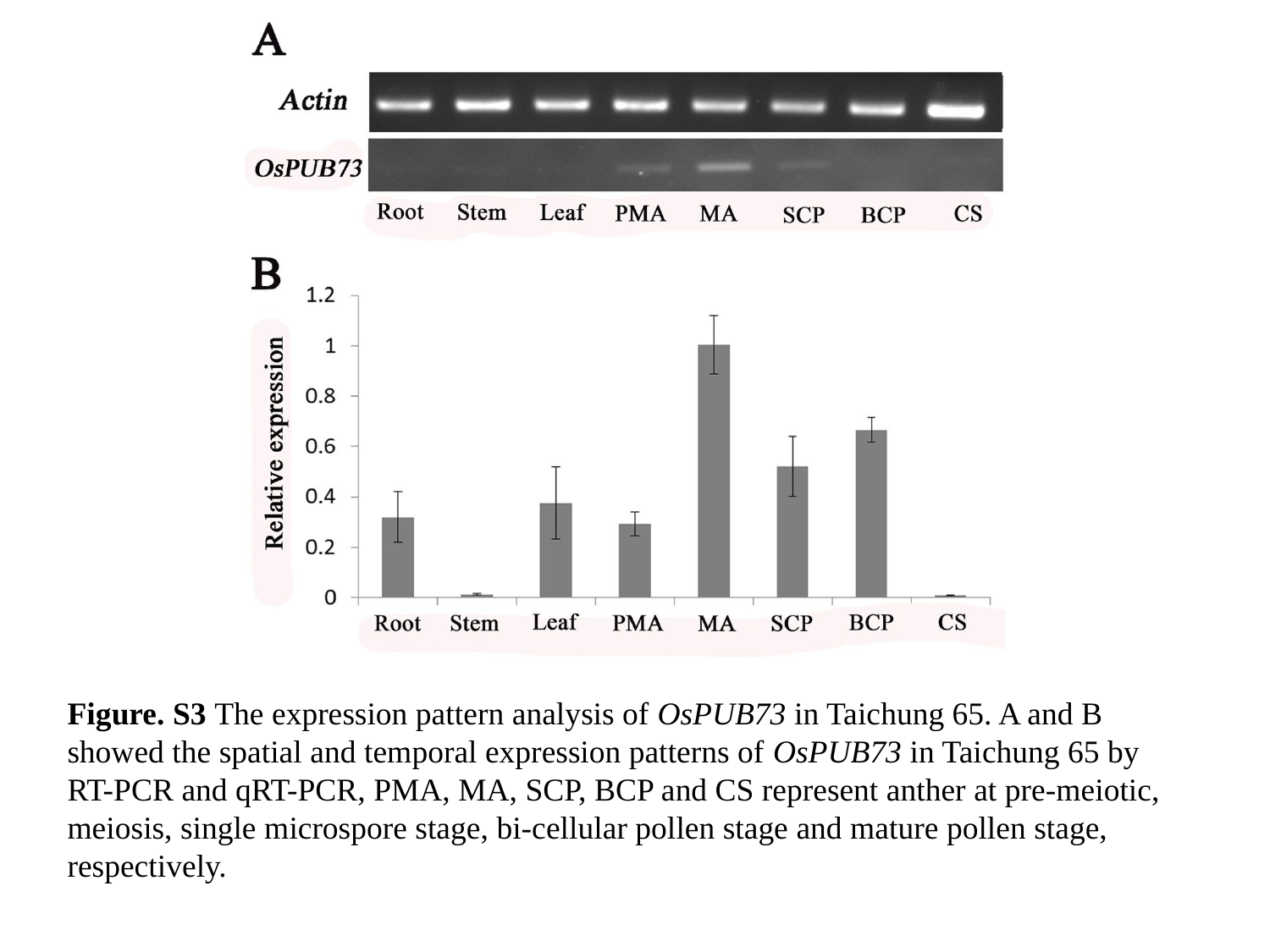

Figure. S3 The expression pattern analysis of OsPUB73 in Taichung 65. A and B showed the spatial and temporal expression patterns of OsPUB73 in Taichung 65 by RT-PCR and qRT-PCR, PMA, MA, SCP, BCP and CS represent anther at pre-meiotic, meiosis, single microspore stage, bi-cellular pollen stage and mature pollen stage, respectively.
